# A transdiagnostic sleep and circadian treatment to improve severe mental illness outcomes in a community setting: study protocol for a randomized controlled trial

**DOI:** 10.1186/s13063-016-1690-9

**Published:** 2016-12-20

**Authors:** Allison G. Harvey, Kerrie Hein, Lu Dong, Freddie L. Smith, Michael Lisman, Stephanie Yu, Sophia Rabe-Hesketh, Daniel J. Buysse

**Affiliations:** 1 Department of Psychology, University of California, 2205 Tolman Hall #1650, Berkeley, CA 94720-1650 USA; 2 Alameda County Behavioral Health Care Services, Oakland, CA USA; 3 University of Pittsburgh, Pittsburgh, PA USA; 4 Department of Education, University of California, 2205 Tolman Hall #1650, Berkeley, CA 94720-1650 USA

**Keywords:** Transdiagnostic, Sleep, circadian, Severe mental illness, Dissemination

## Abstract

**Background:**

Severe mental illness (SMI) is common, chronic and difficult to treat. Sleep and circadian dysfunctions are prominent correlates of SMI, yet have been minimally studied in ways that reflect the complexity of the sleep problems experienced. Prior treatment studies have been disorder-focused—they have treated a specific sleep problem in a specific diagnostic group. However, real life sleep and circadianproblems are not so neatly categorized, particularly in SMI where features of insomnia overlap with hypersomnia, delayed sleep phase and irregular sleep-wake schedules. Accordingly, the aim of this studyprotocol is to test the hypothesis that a Transdiagnostic Intervention for Sleep and Circadian Dysfunction (TranS-C) will improve functional impairment, disorder-focused symptoms and sleep and circadian functioning. Participants across DSM diagnoses and across common sleep and circadian problems are eligible. The elements of TranS-C are efficacious across SMI in research settings with research-based providers. The next step is to test TranS-C in a community setting. Accordingly, this study is being conducted within Alameda County Behavioral Health Care Services (ACBHCS), the Community Mental Health Centre (CMHC) for Alameda County.

**Methods/design:**

120 adults diagnosed with SMI and sleep and circadian dysfunction within ACBHCS will be randomly allocated to TranS-C (*n* = 60) or 6-months of Usual Care followed by Delayed Treatment with TranS-C (UC-DT; *n* = 60). TranS-C is modularized and delivered across eight to twelve 50-minute, weekly, individual sessions. All participants will be assessed before and immediately following treatment and again 6 months later. Primary analysis will examine whether TranS-C significantly improves functional impairment, disorder-specific symptoms and sleep and circadian functioning, relative to UC-DT. Exploratory analysis will examine whether improvements in sleep and circadian functioning predict reduction in functional impairment and disorder-specific symptoms, and whether the intervention effects are mediated by improved sleep and circadian functioning and moderated by previously reported risk factors (demographics, symptom severity, medications, psychiatric and medical comorbidity).

**Discussion:**

This trial tests an important and understudied mechanism—dysregulated sleep and circadian rhythms—in SMI, a novel transdiagnostic treatment approach, in a community setting so as to contribute to the goal of bridging the gap between research and practice.

**Trial registration:**

ClinicalTrials.gov identifier: NCT02469233. Registered on 9 June 2015.

**Electronic supplementary material:**

The online version of this article (doi:10.1186/s13063-016-1690-9) contains supplementary material, which is available to authorized users.

## Background

The number of people receiving a treatment for severe mental illness (SMI) has risen [1–3], and health care expenditures have skyrocketed [2]. Yet, less than half of the treatments delivered have an evidence base [4–6], and there is no evidence that available treatments decrease disability [7]. Instead, the prevalence of mental illness is increasing [8–10]. Accordingly, providing the large number of people with an SMI access to evidence-based treatments requires fundamentally new approaches [11, 12].

One relatively new approach is to target research and treatment at a transdiagnostic process. A *transdiagnostic process* is defined, in mental health, as a clinical feature in common across more than one mental illness [13–16]. The advantage of targeting research and treatment at a transdiagnostic process is threefold. First, if a transdiagnostic process contributes to the maintenance of symptoms across multiple disorders, then one approach is to develop treatments based on the process rather than on the large number of discrete disorders currently listed in the *Diagnostic and Statistical Manual of Mental Disorders, Fifth Edition* (DSM-5) [14]. Second, comorbidity is the norm [17, 18]. Hence, a significant clinical dilemma is which disorders to prioritize for treatment [14]. Treating transdiagnostic processes provides one path forward [13, 14]. Third, a transdiagnostic approach may reduce the heavy burden on clinicians, who must learn multiple disorder-focused protocols, by focusing on common theoretical underpinnings and interventions [13].

Sleep and circadian dysfunction has been highlighted as a biologically [19] and theoretically [20] plausible transdiagnostic contributor to SMI [13], and a transdiagnostic treatment for sleep and circadian disturbance has been proposed [21]. The present study protocol tests the hypothesis that the Transdiagnostic Intervention for Sleep and Circadian Dysfunction (TranS-C) for participants with SMI will improve functional impairment, disorder-focused symptoms, and sleep and circadian dysfunction.

### Why is sleep and circadian dysfunction important in SMI?

First, sleep and circadian dysfunction coexists with, predates, and predicts SMI. Insomnia, hypersomnia, delayed sleep phase, and irregular sleep-wake schedules are commonly comorbid with SMI [22–26]. The rate of insomnia across DSM disorders is approximately 50% [25]. The rate of hypersomnia is as high as 75% across the mood disorders [26]. These problems often persist even with best practice treatment for SMI [27–31]. Across multiple longitudinal studies, sleep and circadian dysfunction predicts and predates the onset and worsening of SMI symptoms [32–41]. Second, sleep and circadian dysfunction contributes to vicious cycles of mutually reinforcing symptoms in SMI, including emotional dysfunction [42, 43], poor health [44, 45], cognitive dysfunction [46, 47], and behavior problems [48, 49]. Third, sleep and circadian dysfunction is modifiable in SMI. Cognitive behavioral therapy for insomnia (CBT-I) effectively treats insomnia that is comorbid with a wide range of SMIs, including major depressive disorder [50], posttraumatic stress disorder [51–54], schizophrenia [55], bipolar disorder [56, 57], alcohol dependence [58], and mixed SMI [59–61]. There is evidence that these gains are well-maintained following the cessation of treatment [62]. There is also evidence that treating insomnia improves the symptoms of the comorbid disorder [50, 51, 55, 63]. In our study, we will test a treatment that addresses an important and understudied transdiagnostic mechanism—dysregulated sleep and circadian rhythms—in SMI. It also addresses an understudied conceptual framework, namely that sleep and circadian dysfunction contributes to vicious cycles of escalating vulnerability and increased risk in SMI.

The majority of prior studies have been disorder-focused—they have been designed to treat a specific sleep problem (e.g., insomnia) in a specific diagnostic group (e.g., depression, posttraumatic stress disorder). Real-life sleep and circadian problems are not so neatly categorized, particularly in SMI. Indeed, insomnia can overlap with hypersomnia [26, 33, 64–67], delayed sleep phase [68], and irregular sleep-wake schedules [28]. In a prior study, CBT-I was modified to address the broader range of sleep dysfunctions in SMI [56, 57], adding elements from interpersonal and social rhythm therapy [69, 70], chronotherapy [71], and motivational enhancement [72–74]. Strong results reported by other groups [75] and advice from dissemination experts [76, 77] led us to develop a modularized transdiagnostic treatment [21].

### Real-world setting: community mental health centers

There has been ample testing of sleep and circadian interventions in specific disorders in research settings with research-based providers. Hence, the next step is to conduct an “efficacy in the real world” study *in* community settings with community-based providers while maintaining the high level of control necessary to establish internal validity. This next step is important because there is currently a 15- to 20-year lag between treatment discovery and incorporating new treatments into routine practice [12, 78]. Initial results indicate that providing CBT-I in various real-world settings is effective [79–81].

### Sleep health

The sleep health framework [82] underpins and guides TranS-C. The sleep health framework prompts a shift from a singular focus on the identification and treatment of sleep disorders to a health promotion perspective, which emphasizes universal attributes of sleep that can be optimized to promote well-being. The sleep health framework encourages sleep improvement along six dimensions that have been linked to mental and physical health outcomes [82]. The dimensions are (a) regularity of sleep and waking up; (b) satisfaction with sleep or sleep quality; (c) alertness during waking hours or daytime sleepiness; (d) appropriate timing of the patient’s sleep within a 24-h day; (e) sleep efficiency (i.e., the ability to sleep for a large percentage of the time in bed), as indicated by ease of falling asleep at the beginning of the night and the ease of returning to sleep after awakenings across the night; and (f) sleep duration, which is the total amount of sleep obtained by the patient per 24 h.

The main aim of this study protocol is to evaluate the effects of TranS-C vs. usual care followed by delayed treatment with TranS-C (UC-DT) on functional impairment, disorder-focused symptoms, and sleep and circadian function in participants receiving treatment for SMI in a community mental health center. The hypothesis tested is that TranS-C will be superior to UC-DT at posttreatment and 6-month follow-up for functional impairment, disorder-focused symptoms, and sleep and circadian function.

In exploratory analyses, we will address (a) whether improved sleep and circadian functioning predicts reduced functional impairment and disorder-focused symptoms, regardless of treatment condition; (b) whether improved sleep and circadian functioning mediates the intervention effects (TranS-C vs. UC-DT) on functional impairment and disorder-focused symptoms; and (c) whether intervention effects are moderated by previously reported risk factors.

## Methods/design

### Study design and setting

**Fig. 1:**
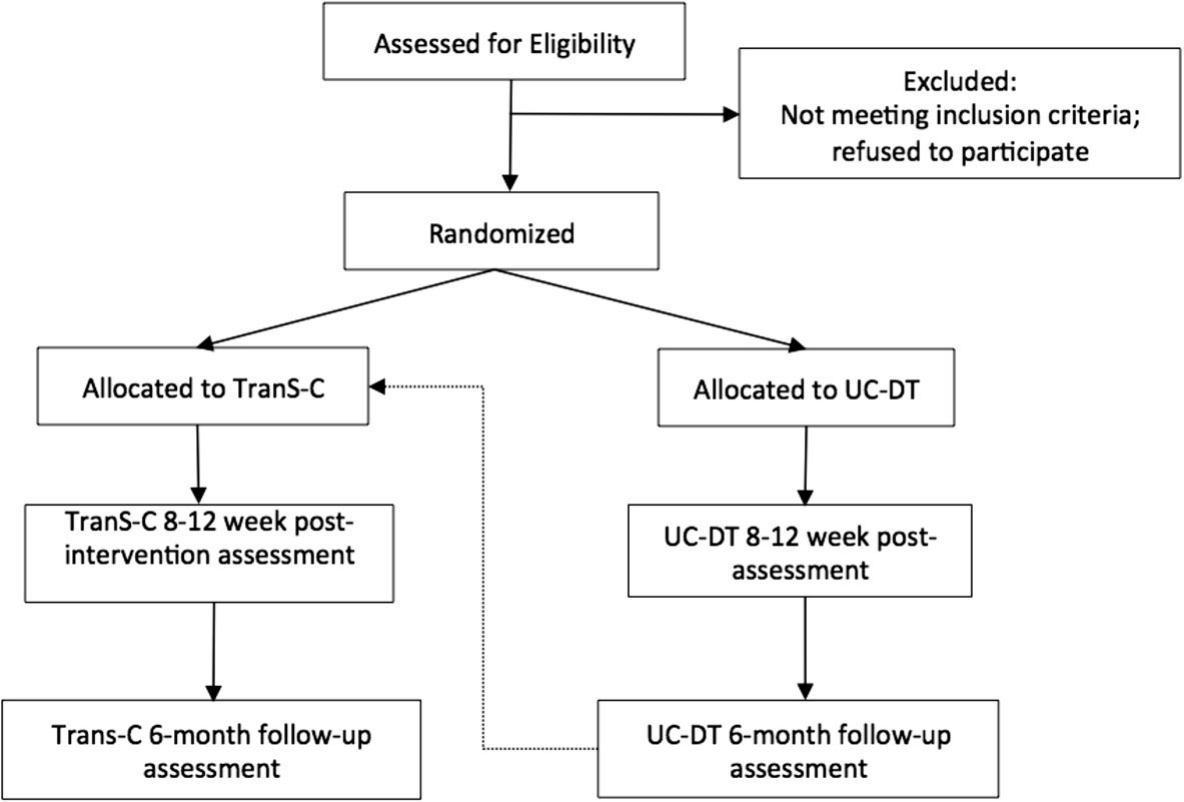
Flowchart of study design

We are conducting a prospective randomized controlled study. Adults (*n* = 120) who meet criteria for SMI and sleep and circadian dysfunction will be randomly assigned, in a 1:1 parallel-group design, to undergo TranS-C (*n* = 60) or UC-DT (*n* = 60) (see Fig. 1 for study design flowchart). Randomization is stratified by psychosis (yes or no) and age (older or younger than 49 years). Participants will receive a battery of outcome measures pretreatment and again at posttreatment (i.e., 9–14 weeks later) and at 6-month follow-up. Those in the UC-DT group will receive two additional assessments: 9–14 weeks and 6 months into UC-DT. An additional assessment of sleep and circadian function will take place in session 4 for the mediation analysis.

The assessment and therapy teams are blinded to treatment allocation. Randomization will be conducted using a computerized random number generator where the planned stratification randomization is part of the allocation sequence. Only the statistician (LD), the project coordinator, and the assigned therapist know the treatment allocation of each participant. Participants are financially compensated for their time. A Data and Safety Monitoring Board will review the study every 6 months during the active treatment phase. A Standard Protocol Items: Recommendations for Interventional Trials (SPIRIT 2013) checklist (see Additional file 1) and figure (see Table 1) are provided [83].

**Table 1:** SPIRIT checklist

Time point	Study period					
	Enrollment	Pretreatment assessment	Allocation	Postallocation		
				Intervention	Post-Treatment Assessment	6-Month follow-up
Enrollment						
Eligibility screen	X					
Informed consent	X	X				
Allocation			X			
Intervention						
TranS-C				X		
UC-DT^1^						
Data collection						
Demographics		X			X	X
Primary outcomes		X			X	X
Secondary outcomes		X			X	X
Diagnostic measures		X			X	X
Sleep/insomnia history		X				
Medication tracking		X		X	X	X
Credibility/expectancy				X		

*Abbreviations: TranS-C* Transdiagnostic Intervention for Sleep and Circadian Dysfunction, *UC-DT* Usual care followed by delayed treatment with Transdiagnostic Intervention for Sleep and Circadian Dysfunction.^1^Allocation to TranS-C after 6-months of usual care.

For participants who discontinue, the assessment team will endeavor to collect all assessment data, prioritizing the primary outcomes.

### Participants

A total of 120 adults who meet criteria for SMI and sleep and circadian dysfunction are recruited from multiple sites within Alameda County Behavioral Health Care Services (ACBHCS; Alameda County, CA, USA). To date, these sites are Oakland Community Support Center (Oakland), Eden Mental Health Services (San Leandro), Tri City Community Support Center (Fremont), Alameda Support Center (Alameda), Axis Community Health (Pleasanton), La Familia Counseling (Hayward), and multiple board and care homes. Participants are referred via ACBHCS case managers and doctors. The inclusion and exclusion criteria are presented below. To enhance representativeness and generalizability, the inclusion and exclusion criteria are kept to a minimum*.*

#### Inclusion criteria

The inclusion criteria are as follows:

- Aged 18+ years
- English language fluency
- Presence of at least one DSM-5 mental disorder for 12 months
- One or more of the following sleep or circadian problems for 3 months assessed with the
  Sleep and Circadian Problems Interview:
  - ≥30 minutes to get to sleep on three or more nights per week
  - Waking in the middle of the night for ≥30 minutes on three or more nights per week
  - Obtaining <6 h of sleep per night on three or more nights per week. Obtaining more than 9 hours of sleep per 24 hour period (i.e., nighttime sleep plus daytime napping), 3 or more nights per week
  - More than 2.78 h of variability in sleep-wake schedule across 1 week
  - Bedtime later than 2:00 a.m. on three or more nights per week
- Having a guaranteed bed to sleep in for 3 months
- Receiving care for SMI at ACBHCS and consent to regular communications between research team and psychiatrist and/or case manager

#### Exclusion criteria

The exclusion criteria are as follows:

- Presence of an active and progressive physical illness or neurological degenerative disease and/or substance abuse/dependence making participation in the study infeasible
- Current serious suicide risk (assessed by our staff, a case manager, or a psychiatrist) or homicide risk (assessed by our staff, a case manager, or a psychiatrist)
- Night shift work >2 nights per week in the past 3 months
- Pregnancy or breastfeeding
- Not able/willing to participate in and/or complete the pretreatment assessments

For the present study protocol, SMI is operationalized according to U.S. Public Law 102-321 and previous research [84–86] as the presence, for 12 months, of at least one DSM-5-defined mental disorder that leads to substantial interference with one or more major life activities [87].

Sleep apnea and periodic limb movement disorder are often comorbid with insomnia [88, 89], and individuals with these sleep disorders typically have poor sleep habits. Hence, we elected to include these individuals. Indeed, there is already some evidence that these participants benefit from CBT-I [63, 90].

Pharmacotherapy for SMI is a complex undertaking guided both by empirical evidence and by the specific experiences and responses of individual participants. Excluding participants whose medications need to be changed is neither feasible nor representative of clinical practice [91]. Medication use and changes will be recorded. All medication decisions will ultimately rest with the treating physican and participant.

### Measures

In addition to demographics (age, contact information, sex, race/ethnicity, family, education, employment, living arrangements, government assistance, housing), the measures described in the subsections below will be administered.

#### Primary outcomes

Functional impairment is assessed with the Sheehan Disability Scale [92], which is a widely used brief measure. The DSM-5 Cross-Cutting Measure is used as a measure of disorder-focused symptoms. Sleep and circadian function is assessed with the Patient-Reported Outcomes Measurement Information System–Sleep Disturbance (PROMIS-SD) [93, 94] and the Patient-Reported Outcomes Measurement Information System–Sleep-Related Impairment (PROMIS-SRI) [93, 94], which are brief, comprehensive, and well-validated. The PROMIS scales are also administered at session 4 for the mediation analysis.

#### Secondary outcomes

Impairment is assessed with the self-administered version of the World Health Organization Disability Assessment Schedule 2.0 and the 4-question healthy days core module developed by the Centers for Disease Control and Prevention [95]. Disorder-focused symptoms are assessed using the Quick Inventory of Depressive Symptoms [96]; the Alcohol, Smoking and Substance Involvement Screening Test [97]; and the Psychotic Symptoms Rating Scales [98]. Sleep and circadian function is assessed with the daily sleep diary and actigraphy, collected for 7 days at each assessment point. The outcomes to be analyzed using the sleep diary are the mean and variability in sleep efficiency (total sleep time/time in bed × 100), total sleep time (TST), total wake time (TWT), bedtime, wake time, and rise time. In addition, nap duration will be calculated. The actigraphy outcomes to be analyzed are the means and variability for TST and TWT, as well as the daytime activity count. In addition, we will calculate a composite sleep health score [82], which is defined as the sum of scores on six sleep health dimensions: regularity (midpoint fluctuation across the 7-day sleep diary), satisfaction (sleep quality question on PROMIS-SD), alertness (daytime sleepiness question on PROMIS-SRI), timing (mean midpoint across the 7-day sleep diary), efficiency (sleep efficiency based on the 7-day sleep diary), and duration (TST based on 7-day sleep diary). This measure is proposed to capture the complexity of the sleep problems covered by TranS-C.

#### Other measures

The diagnostic measure used for mental disorders is the M.I.N.I. International Neuropsychiatric Interview (MINI) (version 7.0.0, including schizophrenia and psychotic disorders). The MINI is included as an evaluation of the presence of current and past SMI. The MINI was developed to meet the need for a simple, short, but accurate structured psychiatric interview. Its validity has been well-supported [99–101]. At each assessment, we will report the number of DSM-5 diagnoses derived from the MINI. The diagnostic measure we will use for sleep disorders is the Duke Structured Interview for Sleep Disorders (DUKE) [102].

Sleep/insomnia history is obtained with the Sleep and Circadian Problems Interview, which is an adapted version of the Insomnia Interview Schedule [103]. To improve our ability to identify obstructive sleep apnea, we will supplement the proposed DUKE assessment with the STOP-BANG Questionnaire [104], which is an 8-item screen for obstructive sleep apnea. Both measures are well-validated and widely used. Those suspected to have another sleep disorder will receive nonstudy evaluation/treatment and will not be excluded. At each assessment, we will report the number of sleep diagnoses derived from the DUKE. The medication tracking log will be used to record the treatments patients are receiving. Treatment credibility/expectancy is administered at session 2 via the Credibility/Expectancy Questionnaire [105, 106].

### Treatments

#### TranS-C

TranS-C is administered by master’s-level therapists hired within the University of California, Berkeley, for this study, who travel between the ACBHCS clinic sites. As participants move through the treatment at different rates, TranS-C is provided in eight weekly 50-minute sessions. If patients need additional sessions to cover the treatment, up to four additional sessions may be offered (i.e., maximum of 12 sessions). TranS-C includes four cross-cutting interventions featured in every session; four core modules that apply to the vast majority of participants; and seven optional modules used less commonly, depending on the presentation. Table 2 summarizes the approach.

**Table 2:** Summary of the Transdiagnostic Intervention for Sleep and Circadian Dysfunction

Cross-cutting modules introduced in sessions 1-3 (and featured in all sessions thereafter)	Module Topics in TranS-C	Treatment module
Case formulation	Establishing regular sleep-wake times	Core module 1, part a
Education	Learning a wind-down routine	Core module 1, part b
Behavior change and motivation	Learning a wake-up routine	Core module 1, part c
Goal-setting	Improving daytime functioning	Core module 2
	Correcting unhelpful sleep-related beliefs	Core module 3
	Improving sleep efficiency	Optional module 1
	Reducing time in bed	Optional module 2
	Dealing with delayed or advanced phase	Optional module 3
	Reducing sleep-related worry/vigilance	Optional module 4
	Promoting compliance with CPAP/exposure therapy for claustrophobic reactions to CPAP	Optional module 5
	Negotiating sleep in a complicated environment	Optional module 6
	Reducing nightmares	Optional module 7
	Maintenance of behavior change	Core module 4

*Abbreviations: CPAP* Continuous positive airway pressure, *SMI* Severe mental illness, *TranS-C* Transdiagnostic Intervention for Sleep and Circadian Dysfunction

#### Usual care followed by delayed treatment with TranS-C

The aim of the choice to compare TranS-C with UC-DT is to strike a balance between (a) including a comparison group to test the effectiveness of TranS-C in community settings, information critical to determining the potential of TranS-C for broader dissemination; and (b) ensuring that all participants receive what is hypothesized to be a more active treatment (TranS-C). Usual care in ACBHCS starts with a case manager who coordinates care and refers each client for a medication review and to various rehabilitation programs (e.g., health care, housing, nutrition, physical activity, finding a job, meditation group, tobacco cessation group, peer monitoring). The content of usual care will be monitored using the medication and other treatment tracking logs. As depicted in Fig. 1, at the end of 8 months in UC-DT, the participants will receive 8 to 12 sessions of TranS-C.

#### Treatment implementation and monitoring

Clinicians attend a one-day workshop, use a treatment manual and receive weekly supervision to standardize treatment administration. Treatment sessions are audiotaped, and a random selection are rated using the Cognitive Therapy Scale [107], which is a measure of general intervention skills and CBT-specific skills.

### Data analysis

#### Preliminary data evaluation

Missing or aberrant data will be verified. Data will be audited for quality and completeness. We will detect outliers by evaluation of distributions and ensure that assumptions of planned analyses are met. Prior to hypothesis testing, appropriate statistical tests will be used to examine baseline differences between groups (e.g., race/ethnicity, age, sex, education, employment, psychiatric and medical comorbidities). These tests will not be used to select covariates in the primary intention-to-treat analysis [108–110]. Instead, the potential influences of baseline differences will be evaluated as moderators (described below).

#### Management of missing data

Recruiting 120 participants allows for attrition as per the power calculation below. In longitudinal analyses, we will use all available data and produce valid inferences if attrition depends on treatment group or on previous outcomes for the same participant [111]. If dropout is related to other variables, these data will be included as predictors to reduce any bias due to nonrandom missing data.

#### Main aim

TranS-C will be superior to UC-DT for reducing functional impairment and disorder-focused symptoms, and for improving sleep and circadian functioning, at posttreatment and 6-month follow-up on primary outcomes. To address these aims, we will test whether there are differences in the mean trajectories across time points between TranS-C and UC-DT using hierarchical linear modeling (HLM) [112–114]. The first-level equation will represent within-person variation and will include time indicators (or dummy variables) as predictors (posttreatment, 6-month follow-up assessment, with baseline as a reference). The second-level equation represents between-person variation in the intercept and coefficients of the time indicators, and will include a dummy variable for arm (TranS-C vs. UC-DT) as the predictor variable. Interactions between arm and time indicators will be retained only if found to be significant at the 5% level. A significant interaction between arm and a time indicator suggests that there are different trajectories of change in outcome across time for each arm, and will be displayed as a graph to interpret the interaction.

#### Exploratory analyses

For the exploratory analyses, we will use only the primary outcome measures for impairment, disorder-focused symptoms, and sleep and circadian function:

1. *Do improved sleep and circadian functioning predict reduced functional impairment and disorder-focused symptoms from baseline to posttreatment?* We will use HLM to test whether changes in sleep and circadian functioning predict changes in functional impairment and disorder-focused symptoms from baseline to posttreatment.
2. *Do improved sleep and circadian functioning mediate intervention effects on primary outcomes?* We will conduct mediation analysis using product of coefficients, a powerful method for estimating indirect effects [115]. The mediator will be sleep and circadian functioning measured at session 4, and outcomes will be functional impairment and disorder-focused symptoms measured at posttreatment.
3. *Are intervention effects moderated by risk factors, including demographics, symptom severity, medications, and psychiatric and medical comorbidities?* We will conduct moderation analysis by testing a three-way interaction of treatment arm, time, and the moderator variable in the second-level equation using the HLM model described above [116, 117]. A significant coefficient for the arm × time × moderator interaction would indicate a moderating effect and will be followed with graphs to interpret the modification [116].

A statistical significance level of 0.05 will be used throughout.

#### Power analysis

Average effect size across outcomes akin to those proposed were drawn from prior research [56, 79, 80] (0.60). Using G*Power 3.1.7 software, 80% power, and a two-tailed alpha of 5%, we will need 92 participants to detect group differences. An additional 30% to account for potential attrition yields 120 participants.

## Discussion

The study protocol addresses several research priorities. First, this study provides a test of a treatment that addresses an important and understudied mechanism—dysregulated sleep and circadian rhythms—in SMI. It also addresses an understudied conceptual framework, namely that sleep and circadian dysfunction contributes to vicious cycles of escalating symptoms, vulnerability, and risk in SMI. Second, this study provides a test of a transdiagnostic treatment designed to treat a wide range of sleep and circadian problems experienced by adults with a wide range of SMIs. As such, the study contributes to the goal of developing interventions that use broad, dimensional approaches to assessment and intervention [118, 119]. Third, the sleep health framework [82] that underpins the approach is relatively new. This approach emphasizes the identification and treatment of sleep disorders as well as the universal attributes of sleep that can be optimized to promote well-being. Fourth, the modular design of TranS-C, as well as the personalized behavioral “prescriptions,” are responsive to the calls to “develop a personalized approach to the diverse needs and circumstances of people with mental illness” [120, page 128]. Finally, this study will be conducted in a community setting. Conducting the study in a community setting contributes to the goal of bridging the gap between research and practice and testing treatments in the community. If the study is successful, this research will establish the potential for widespread dissemination of TranS-C to improve SMI outcomes in the community.

### Trial status

The trial is funded for 4 years. A ‘skeleton’ research staff team started setting up the study in February 2015. Patients began to be randomized in July 2015. The treatment phase will be completed in July 2018. Final outcome assessments will be complete by January 2019. As of November 2016, 70 participants of the required 120 had been enrolled.

## Additional files

Additional file 1: Completed SPIRIT checklist of recommended items to address in a clinical trial protocol. (PDF 109 kb)
Additional file 2: Consent form. (PDF 63 kb)

## Abbreviations

ACBHCS: Alameda County Behavioral Health Care Services
CBT-I: Cognitive behavioral therapy for insomnia
CPAP: Continuous positive airway pressure
DSM-5: *Diagnostic and Statistical Manual of Mental Disorders, Fifth Edition*
DUKE: Duke Structured Interview for Sleep Disorders
HLM: Hierarchical linear modeling
MINI: M.I.N.I. International Neuropsychiatric Interview
PROMIS-SD: Patient-Reported Outcomes Measurement Information System–Sleep Disturbance
PROMIS-SRI: Patient-Reported Outcomes Measurement Information System–Sleep-Related Impairment
QIDS: Quick Inventory of Depressive Symptomatology
SMI: Severe mental illness
SPIRIT: Standard Protocol Items: Recommendations for Interventional Trials
TAU: Treatment as usual
TranS-C: Transdiagnostic Intervention for Sleep and Circadian Dysfunction
TST: Total sleep time
TWT: Total wake time
UC-DT: Usual care followed by delayed treatment with Transdiagnostic Intervention for Sleep and Circadian Dysfunction
