# Supplementary material for: A transdiagnostic sleep and circadian treatment to improve severe mental illness outcomes in a community setting: study protocol for a randomized controlled trial
Source: Trials. 2016 Dec 20;17:606. doi: 10.1186/s13063-016-1690-9 (PMC5175375; doi:10.1186/s13063-016-1690-9)
Supplement: Additional file 2: — Consent form. (PDF 63 kb) [file 13063_2016_1690_MOESM2_ESM.pdf]

UNIVERSITY OF CALIFORNIA, BERKELEY

BERKELEY • DAVIS • IRVINE • LOS ANGELES • RIVERSIDE • SAN DIEGO • SAN FRANCISCO SANTA BARBARA • SANTA CRUZ

Golden Bear Sleep & Mood Research Clinic
3210 Tolman Hall #1650
Berkeley, CA 94720-1650
TEL: (510) 643-3797
FAX: (510) 642-5293
EMAIL: gbsmrc@berkeley.edu

Consent to Participate in a SleepTreatment Research Study

My name is Professor Allison Harvey. I am a faculty member in the Department of Psychology, University of California, Berkeley. I would like to invite you to take part in the research that is underway. This study is a collaboration between Alameda County Behavioral Health Care Services (ACBHCS) and our team at the University of California, Berkeley. The aim of this study is to find out if improving sleep can help your mental health. We are hoping that this research will help establish a novel mental health treatment for psychiatric conditions.

Description of the research study

If you agree to take part in this research, we will invite you to complete an interview. The interview will be conducted in a private room at ACBHCS. During this interview, we will ask you questions about your medical and psychological history, as well as some sensitive and personal questions about some of your other behaviors. Additionally, we will ask you to fill out a number of questionnaires and to complete some tasks. You do not have to answer any questions you do not feel like answering. The interview will take approximately 3 hours. You may take breaks during the interview, and we will provide snacks. After the interview, we will ask you to take a call from us to collect some information about your sleep each day for one week and wear an actiwatch for one week.

With your permission, these interviews will be audio-recorded. If you are eligible for and choose to enroll in the study, the sleep coaching sessions will also be audio-recorded, with your permission. This is done so that the lead clinical psychologist can ensure the interviews and sleep coaching sessions are completed to the highest standards. The audio recordings are encrypted and stored on a password-protected computer, which means that no one can listen to them except for project staff.

If it appears that you might benefit from our treatment, you will be invited to participate in the study. If you agree to participate, you will be randomly assigned to one of two groups. While both groups will receive the same sleep coaching, one group begins sleep coaching immediately after the first interview and the other group will be asked to complete two more interviews 2 months and 8 months after the first interview, before beginning sleep coaching. The content of these interviews is similar to the first interview and will take approximately 2-3 hours.

The delayed treatment group plays an important role in the research. Participants that are waiting eight months to begin treatment will have the option of attending monthly socials with other participants who are also waiting to begin.

During the sleep coaching, you will be attending a 25-50 minute session, once per week, for 8-12 weeks, in one of the private rooms at ACBHCS. In each session, the sleep coach will work together with you to

determine what is causing the sleep problems negatively affecting your mental health. Then, with your sleep coach, you will develop ways to change those things that are contributing to these problems. This study will not give you medication and all parts of the treatment involve talking and working with your sleep coach.

Once you complete the sleep coaching sessions, we will interview you again to see how you are doing after the sleep coaching. We would like to do this on two occasions; 1 week after sleep coaching has finished and again 6 months after sleep coaching has finished. These interviews will involve the same activities as the first interview and will take approximately 2-3 hours. You may always take breaks during the interview, and we will provide snacks.

Summary of total time commitment

All participants will attend:

Pre-sleep coaching interview: maximum of 3 hours for interview and 5 minutes per day for 7 days to collect sleep diary

Treatment sessions: 8-12 sessions (25-50 minutes each)

1 week post-sleep coaching interview: maximum of 3 hours for interview and 5 minutes per day for 7 days to collect sleep diary

6 months post-sleep coaching interview: maximum of 3 hours for interview and 5 minutes per day for 7 days to collect sleep diary

Some participants will attend 2 additional interviews:

8 weeks after first interview: maximum of 3 hours for interview and 5 minutes per day for 7 days to collect sleep diary

8 months after first interview: maximum of 3 hours for interview and 5 minutes per day for 7 days to collect sleep diary

Risks

Occasionally a participant can find it upsetting to talk about the difficulties they are experiencing relating to their mental health. You may choose to not answer or skip any question you do not wish to answer, and you have the right to discontinue your involvement in the study at any time.

Benefits

It is hoped that your participation in this study will help improve your mental health symptoms and that this research will help improve available treatments for people experiencing mental health difficulties.

Compensation/Costs

In gratitude of your time and to cover any travel expenses you may have incurred, we would like to offer you:

At each interview:

- $10 gift card for completing a 7-day sleep diary
- $10 gift card and a "Sleep Team" wristwatch for the return of the actiwatch after wearing it for 7 days
- $30 gift card for completing the interview

At the post-sleep coaching interview:

- "Berkeley Sleep Team" t-shirt

For all interviews and sleep coaching sessions:

- Assistance with transportation to Oakland Community Support Center

Confidentiality

All of the information that I obtain from you during the research will be kept as confidential as possible. All data collected from you will be identified in study records by a code number and your individual identity that is linked with the code will be stored separately in a locked file and/or encrypted spreadsheet. I will encrypt the audio recordings and store them on a password-protected computer. I will not use your name or other identifying information in any reports of the research without your additional consent. After this research is completed, I may save the recordings and my notes for use in future research by myself and others in the research group. However, the same measures described above will apply to future storage and use of the materials. Note that while there is a small chance that the confidentiality of the information collected could be compromised, we will take precautions to minimize this risk.

We will keep your study data as confidential as possible, except for certain information that we must report for legal or ethical reasons, such as child abuse, elder abuse, or the intention to hurt yourself or others.

Rights and Other Information

Your participation in this research is voluntary. You are free to withdraw your consent and discontinue participation in this study at any time. If you participate throughout this study, you may refuse to answer any question(s) that might make you feel uncomfortable.

I would like you to be aware that the sleep study has special criteria for selecting people to participate, so some people may be invited to participate, and it may be the case that some who are interviewed are not a good fit for the study. Whether or not you are invited to be a sleep study participant, we would like to give you a gift card for your interview time, and we also will be available for any questions you have about sleep. With your permission, information about you will be kept because it will help us with the research, but identifiable information about participants is never given in publications.

Authorization for Release/Exchange of Mental Health and Medical Information

I authorize the Berkeley Sleep Team at the University of California, Berkeley, Alameda County Behavioral Health Care Services Oakland Community Support Center, and Lifelong Medical Care to release/exchange information pertaining to mental health diagnosis or treatment, information pertaining to drug and alcohol abuse diagnosis or treatment, and/or other medical information for the purpose of conducting research-related treatment (sleep coaching).

This Authorization to release mental health and medical information is voluntary and may be revoked at any time. The revocation must be in writing, signed by you or your client representative, and delivered to the Berkeley Sleep Team Office at Oakland Community Support Center, 7200 Bancroft Avenue Suite 125-A, Oakland, CA 94605. The revocation will take effect when the Berkeley Sleep Team office receives it, except to the extent the Berkeley Sleep Team or others have already relied on it.

Unless otherwise revoked, this Authorization expires 18 months after the date of you signing the form.

______________________________ ______________________________

Signature Date Time

Consent to Be Invited to Sleep Team Activities

The **Health Insurance Portability and Accountability Act, HIPAA**, protects your privacy. This means that we need your permission to invite you to activities related to Sleep Team, such as monthly socials for the participants in the delayed treatment group or Lunch & Learn events. If you consent to participate in these events, please be aware that it is entirely your choice as to whether or not you identify to others that you are receiving or have received sleep coaching.

Please initial one:

____*I consent* to be invited to activities related to the sleep study.

____*I do not consent* to be invited to activities related to the sleep study.

Questions

If you have any questions about the research, you may call me, Allison Harvey, at (510) 643-3797.

If you have any questions about your rights or treatment as a participant in this research project, please contact the University of California at Berkeley's Committee for Protection of Human Subjects at (510) 642-7461, or e-mail: subjects@berkeley.ed

SIGNATURE OF RESEARCH SUBJECT

If you agree to take part in the research, please sign the form below. Please keep the other copy of this agreement for future reference.

Name:________________________________ Date:____________

Signature:________________________________ Date:____________

SIGNATURE OF INVESTIGATOR OR DESIGNEE

In my judgment the subject is voluntarily and knowingly giving informed consent and possesses the legal capacity to give informed consent to participate in this research study.

Name:________________________________ Date:____________

Signature:________________________________ Date:____________
